# Development and validation of the clinical report form for nodular thyroid pathologies

**DOI:** 10.20945/2359-3997000000534

**Published:** 2022-12-01

**Authors:** Vanessa Neto, Sara Esteves-Ferreira, Isabel Inácio, Márcia Alves, Rosa Dantas, Teresa Azevedo, Joana Guimarães, Maria Teresa Herdeiro, Alexandra Nunes

**Affiliations:** 1 Universidade de Aveiro Instituto de Biomedicina Departamento de Ciências Médicas Aveiro Portugal Instituto de Biomedicina (iBiMED), Departamento de Ciências Médicas, Universidade de Aveiro, Aveiro, Portugal; 2 Centro Hospitalar do Baixo Vouga Aveiro Portugal Centro Hospitalar do Baixo Vouga (CHBV), Aveiro, Portugal

**Keywords:** Nodular thyroid pathologies, clinical report form, structured interview, cohort characterization

## Abstract

**Objectives:**

The aim of this study is to develop and validate a novel clinical report form in the format of a structured interview to enable the characterization of the Portuguese population of the Baixo Vouga region with different subtypes of nodular thyroid pathologies (NTyPs).

**Materials and methods:**

A structured interview was developed and to the best of our knowledge, this is the first structured interview built and validated for that purpose in Portugal.

**Results:**

This structured interview enables the identification of possible correlations between each subtype of nodular lesions and sociodemographic data, consumption habits and lifestyle, endocrine history, and family predisposition.

**Conclusion:**

The novel structured interview will simultaneously, enable a detailed characterization of the group of patients with nodular thyroid lesions and will support future metabolomic studies.

## INTRODUCTION

Nodular thyroid disease, a pathology described by the American Thyroid Association as “discrete lesions within the thyroid gland, radiologically distinct from surrounding thyroid parenchyma,” is one of the most prevalent endocrine diseases all over the world that can result from the development of benign or malignant thyroid nodules (1–3). Although the majority of these nodules are benign (about 90%), there are also malignant nodular lesions, which manifest as thyroid cancer; these are the most clinically relevant lesions that require further health care (4–7).

Over recent decades, the world incidence and prevalence of thyroid cancer have been rising exponentially, with a threefold increase from 1975 to 2009, affecting predominantly female and elderly individuals. This rise is mainly triggered by an increase in the detection of this pathology through the more widespread use of diagnostic techniques such as imaging techniques and fine-needle aspiration (FNA) biopsies (8,9). In Portugal the situation is no different; nodular thyroid disease is considered the third most common type of cancer in women, with an incidence higher than the estimated global and European levels for women (10,11). It is known that in Portugal, in 2016, thyroid cancer was the most frequent cancer in women, with an incidence of 22.6/100000 (10).

Thyroid cancer can manifest as differentiated thyroid carcinomas (papillary, follicular and Hürthle cell carcinomas) or as undifferentiated thyroid carcinoma (anaplastic thyroid carcinoma), both types originating from thyroid follicular epithelial cells, and also as sporadic or hereditary medullary thyroid carcinoma, originating from parafollicular C-cells (5,12). Morbimortality associated with thyroid cancer derives mostly from complications of extrathyroidal extension, which occurs in about 10%-15% of patients; however, as a general rule, this disorder is associated with a good long-term prognosis (1,13). These extrathyroidal extension complications can result from either invasion of adjacent structures, namely the strap muscles (53%), recurrent laryngeal nerve (47%), trachea (37%), esophagus (21%) and larynx (12%), which generally culminates in the manifestation of compressive symptoms (dysphonia, dysphagia, dyspnea and upper airway obstruction, among others), or distant metastases affecting organs such as lungs (75%), bone (45%) and brain (6.8%), with 25% of patients presenting more than one affected organ (1,13–16).

Although the causes of the development of thyroid nodules are not always exactly known, it is well-known that factors of different natures (environmental, genetic and intrinsic to the individual) may be involved in this process (3,17). Sociodemographic aspects (age, gender, body mass index, place of residence, occupation), consumption habits and lifestyle (eating and smoking habits, practice of physical exercise, exposure to radioactive substances) and clinical aspects (patient’s clinical history, genetic predisposition to thyroid pathologies) are closely related to the development of nodular thyroid lesions and thus are excellent tools enabling the characterization of the population with these pathologies (2,3,13,18–20).

The aim of this work is the elaboration and validation of a clinical report form (CRF) in the form of a structured interview that encompasses the various domains mentioned above to enable the characterization of the population of the Baixo Vouga region with the various subtypes of NTyPs and, consequently, the verification of the existence of possible correlations between each subtype of these nodular lesions and the different domains of questions covered by the interview.

## MATERIALS AND METHODS

### Ethics

To execute this project, an ethical approval for the study was obtained in December 2019 from the Ethics Committee for Health of *Centro Hospitalar do Baixo Vouga* (CHBV) (Aveiro, Portugal).

### Study population and settings

Aveiro is a west-coast district located in the central region of Portugal comprising 19 municipalities. One of the National Health Service hospitals serving the population of Aveiro is CHBV, a hospital that provides health care to the residents of 9 municipalities, namely Águeda, Albergaria-a-Velha, Aveiro, Estarreja, Ílhavo, Murtosa, Oliveira do Bairro, Sever do Vouga and Vagos. The area of influence of CHBV includes a population of approximately 285,000 inhabitants – about 2.75% of the total Portuguese population – of which approximately 52.20%, that is, the majority, are female and 47.80% are male.

For this study, the target population included patients with one or more nodular thyroid lesions who had undergone the FNA biopsy technique and who were able to provide informed consent for inclusion in the study. On the other hand, no exclusion criteria were defined, with the exception of the obligation to comply with all the inclusion requirements mentioned above, since one of the objectives of this study was to collect as many samples as possible so that these could later be characterized and classified.

The health professionals of the Endocrinology Service of CHBV played a preeminent role throughout the various stages of the study, including in recruiting participants. To do so, they selected patients exclusively based on clinical data and were also responsible for giving patients all the information about the scope and the objective of the study that was essential for the process of obtaining informed consent.

### Item generation and structured interview design

After selection of the cohort of patients for this study, the structured interview was administered. To develop this clinical document in accordance with all the basic standards necessary for its design and applicability in clinical research and to understand what relevant information should be covered by each of its sections, a vast literature review was carried out through scientific publication search platforms (PubMed and ScienceDirect).

### Content validation and face validity

To validate the content and determine the face validity of the structured interview to be administered, all statements were evaluated not only regarding the clinical terminology used, but also regarding their meaning, formulation and clarity so that informative responses could result from them, avoiding the emergence of misunderstandings (21).

This validation process was performed by a multidisciplinary team consisting of 11 experts, namely by 6 endocrinologists, 4 biomedical researchers and 1 pharmacist, from the University of Aveiro and CHBV.

This interview will be administered by the endocrinologists to each patient selected in the previous phase when the patients visit the Endocrinology Service of CHBV. This strategy makes it possible to fill in all the requested information, including that unknown by the patient, for example, through access to the hospital’s database, thus resulting in the absence of incomplete interviews and a high response rate (21).

## RESULTS

**Figure 1:**
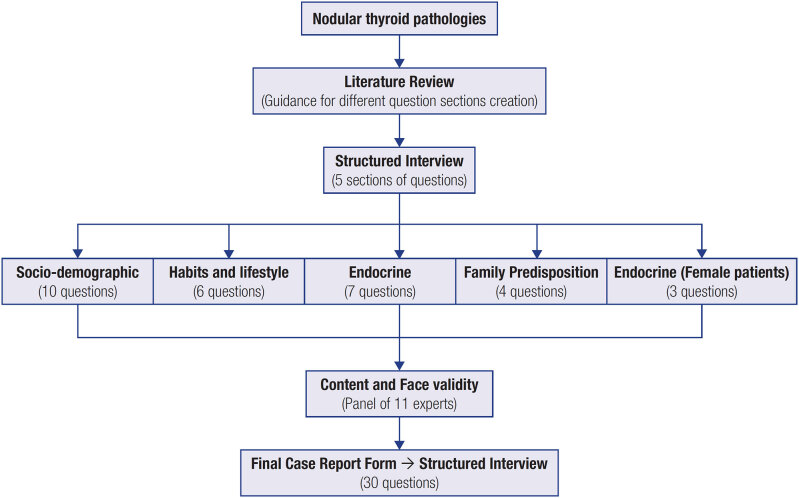
Schematic representation of the various steps involved in the development and validation of a CRF, to be administered as a structured interview, that would enable the characterization of the population of Baixo Vouga region with various NTyP.

Several steps enabled the elaboration of a CRF, to be administered as a structured interview, that would enable the characterization of the population with NTyP at the Endocrinology Service of CHBV (Figure 1). After an extensive literature review to compile all the rules for designing an interview that could be applied in clinical practice and to obtain all the relevant information to be addressed in the different sections, a structured interview, to be administered by endocrinologists to each of the patients recruited, was prepared.

The structured interview (Table 1) will be administered as a three-page paper document, easy to complete, consisting of a total of 30 questions written in Portuguese, either open-answer or closed-answer (multiple choice and multi-item scale questions), which are subdivided into five distinct sections:

- Section 1 – Sociodemographic data (10 questions about the patient’s personal data);
- Section 2 – Consumption habits and lifestyle (6 questions about food practices, physical activity, medication and exposure to harmful substances);
- Section 3 – Examination and endocrine history (7 questions about the clinical history of the patient and his or her close relatives regarding endocrine disorders);
- Section 4 – Family predisposition (4 questions about the clinical history of the patient and his or her close relatives in relation to the occurrence of cancerous disorders);
- Section 5 – Endocrine history (3 questions only for female patients about pregnancy and hormone supplementation methods).

**Table 1:** Structured interview, consisting of 30 questions divided into 5 sections (sociodemographic data, consumption habits and lifestyle, examination and endocrine history, family predisposition, endocrine history of female patients), to be completed by an endocrinologist

Section 1: Sociodemographic data		
Age (years)		
Gender		
	(1) Female	
	(2) Male	
Height (cm)		
Weight (kg)		
Zip code		
Time in actual residence (years)		
Residence area		
	(1) Urban	
	(2) Rural	
Level of completed studies		
	(1) No studies or incomplete primary studies	
	(2) Primary studies	
	(3) Secondary studies	
	(4) Higher education	
Current occupation		
Time of performance in the current occupation (years)		
**Section 2: Consumptions habits and lifestyle**		
Exposure, with consent, to chemical products at work		
	(1) Yes	
	(2) No	
Frequency of food intake of each one of the of the following (dairy products, coffee, tea, eggs, fresh vegetables, canned vegetables, fresh fruit/natural juices, canned fruit, soft drinks, organic food, fast-food, poultry meat, red meat, fresh/frozen fish, canned fish, shellfish, grilled food, smoked food and bottled water)		
	(1) Never	
	(2) 1-3x/month	
	(3) 1-3x/week	
	(4) 4-6x/week	
	(5) Every day	
Smoker		
	(1) Yes	
	(2) No	
	If yes, number of cigarettes per day?	
	If stopped smoking, how long ago did it happened?	
Alcoholic beverages intake		
	(1) Yes	
	(2) No	
If yes, number of glasses per day?		
Physical exercise practice		
	(1) Yes	
	(2) No	
	If yes, how often?	
		(1) 1-3x/month
		(2) 1-3x/week
		(3) 4-6x/week
		(4) Every day
Regular medication		
	(1) Yes	
	(2) No	
	If yes, which one?	
**Section 3: Examination and endocrine history**		
Endocrine system pathology		
	(1) Yes	
	(2) No	
	If yes, which one?	
Conditions that may influence the Endocrine System		
	(1) Yes	
	(2) No	
	If yes, which one?	
Drug/supplement/mineral salt intake for hormonal normalization		
	(1) Yes	
	(2) No	
	If yes, which one?	
Family member with history of thyroid disorders		
	(1) Yes	
	(2) No	
	If yes, which one?	
		Family degree?
		Age?
	Did some treatment?	
		(1) Yes
		(2) No
	If yes, which one?	
Hoarse feeling in the last year?		
	(1) Yes	
	(2) No	
	If yes, how much interfered with daily life?	
		(1) Absolutely nothing
		(2) Little
		(3) Moderately
		(4) Considerable
		(5) Greatly
Swallowing difficulty in the last year?		
	(1) Yes	
	(2) No	
	If yes, how much interfered with daily life?	
		(1) Absolutely nothing
		(2) Little
		(3) Moderately
		(4) Considerable
		(5) Greatly
Breathing difficulty in the last year?		
	(1) Yes	
	(2) No	
	If yes, how much interfered with daily life?	
		(1) Absolutely nothing
		(2) Little
		(3) Moderately
		(4) Considerable
		(5) Greatly
**Section 4: Family predisposition**		
Have you ever had genetic testing?		
	(1) Yes	
	(2) No	
	If yes, and if was obtained a positive result, which was the mutation/syndrome identified?	
**Section 4: Family predisposition**		
Family members with a history of cancer		
	(1) Yes	
	(2) No	
	If yes:	
		Family degree?
		Age at diagnosis?
		Gender?
		Cancer location?
Diagnosed cancer		
		(1) Yes
		(2) No
		If yes, what is the type and location of cancer?
Cancer screenings		
		(1) Yes
		(2) No
		If yes, how often?
**Section 5: Endocrine history (only for female patients)**		
Endocrinology appointment arise after pregnancy?		
		(1) Yes
		(2) No
Take oral contraceptive?		
		(1) Yes
		(2) No
If already in menopause, do any hormone replacement treatment?		
		(1) Yes
		(2) No

## DISCUSSION

To the best of our knowledge, this is the first validated interview for the Portuguese population, and, in the first stage, it will enable the study of NTyP in the area of influence of the CHBV.

The area of influence of the CHBV covers 9 of the 19 districts of the Aveiro district – Águeda, Albergaria-a-Velha, Aveiro, Estarreja, Ílhavo, Murtosa, Oliveira do Bairro, Sever do Vouga and Vagos – and provides health care to about 285,000 residents.

NTyP, an endocrine disease that is quite widespread throughout the world, consists, as the name implies, in small nodular lesions, distinguishable from the remaining thyroid tissue, which can be benign (about 90%) or malignant (between 5% and 10%) (1,3–5). In Portugal, thyroid cancer is the third most common type of cancer in the female population, having even been considered, in 2016, the most common type of cancer in this population, with an incidence of 22.6/100000 (10). Although the incidence of thyroid cancer is more pronounced in the female population, it is in men that there is a higher mortality rate, since this sex is associated with a higher risk of malignancy (3,8,10,11).

Although, at a later stage, patients with thyroid cancer may manifest more complex symptoms resulting from invasion of thyroid adjacent structures or distant metastasis, generally, at an earlier stage, these patients are asymptomatic, which often results in an accidental diagnosis (1,2,8,13). Therefore, in general, the diagnosis of thyroid cancer starts with more elementary diagnostic techniques (physical examination, assessment of the clinical history) and then progresses to the application of more complex diagnostic methods (laboratory blood tests, imaging techniques) (2,3). Despite this huge variety of procedures, the FNA biopsy technique is the preferred diagnostic method for thyroid cancer and is routinely applied in the clinic (3,22).

Thyroid nodules do not always derive from a single *a priori* known cause, however, it is well-known that the interaction between several genetic (family predisposition, mutations and genetic rearrangements), environmental (food consumption, exposure to radioactive substances) and intrinsic factors (age, gender) may be at the base of the evolution of these lesions (3,8,13,17,19).

**Figure 2:**
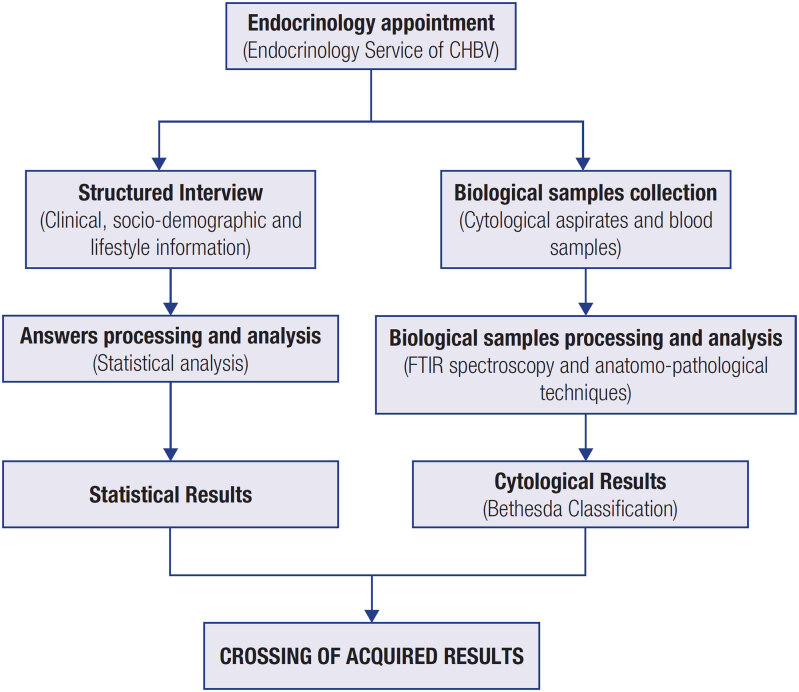
Schematic representation of the procedure workflow followed by our research group, involving all the steps until the crossing of cytological data and interview answers, for the characterization of the cohort of patients from the Endocrinology Service of CHBV.

To characterize the population of the Baixo Vouga region with NTyP, a structured CRF, to be applied in each patient’s appointment as an interview, was developed to verify whether there exists some pattern of association between these diseases and the risk factors mentioned above. During endocrinology visits to the CHBV, selected patients will also submit to collection of cytological aspirates, for the application of the FNA biopsy technique, and blood samples in accordance with standard clinical practice. Subsequently, the interview answers of all patients will be analyzed in detail through the application of statistical methods, while the collected biological samples will be analyzed by Fourier-transform infrared (FTIR) spectroscopy and anatomo-pathological techniques for Bethesda classification. After obtaining the results of the cytological examination of each patient, a comparison between these results and the data acquired during the interviews will be made to verify the existence of any response pattern in the various domains covered by the interview that allows the characterization of patients according to the type of nodular thyroid lesion they present. Figure 2 describes the procedure workflow followed by our research group. The characterized samples will also support the metabolomic study of this type of pathology.

In conclusion, thyroid nodular lesion is one of the most high-incidence and prevalent endocrine disorders in the vast majority of the world, a situation also observed in Portugal. Although the vast diversity of risk factors that may be responsible for its appearance and/or development is evident, namely factors of a genetic, environmental or intrinsic nature, it is not certain which factors are associated with each subtype of nodular thyroid injury or even if the same subtype of nodular lesion in different people is caused by the same set of factors. Therefore, the detailed characterization of the group of patients with NTyP in Portugal is essential; this characterization can be achieved through the administration of the structured interview prepared by our group. In addition to enabling the collection of information about patients and about the pathology itself, this interview may also, in the future, be an asset at the level of research and clinical practice, acting as a tool at the early stage of the process of thyroid cancer diagnosis.
